# An Improved Method of Using Gold Foil

**Published:** 1856-12

**Authors:** 


### Selected Articles.

### AN IMPROVED METHOD OF USING GOLD FOIL.

BY PROF. ARTHUR.

It is now somewhat more than a year since I called the attention of the profession to what I deemed a new method of using gold foil for filling teeth. The method proposed was to roll up the foil, very loosely, in the form of a rope, pass it quickly through the flame of a spirit lamp, cut it up into small pices, and pack it into the cavity to be filled, with sharply serrated instruments, condensing each piece as it is put into place. The object of heating the foil, as I stated, is to give it a quality of adhesiveness not possessed at the time of using, before it is heated, and so affecting its condition, that each piece put into the cavity is made to adhere, closely, to the portions preceding it, although these may have been thoroughly condensed. It was not, as I stated in the article refered to, simply the annealing the gold, at the time of using, that I supposed to be new, as this I knew had been done many years before, but in addition to this, of condensing it thoroughly in small portions, with sharply serrated instruments. I found great advantages from its use in this way, and simply wished to place other operators on the same track I was pursuing myself.

It was immediately declared by a number of members of the profession, that there was nothing new in the method proposed, that it had been adopted and followed by them years before the appearence of my article. Now, I do not mean to question the sincerity of these gentlemen, some of whom occupy high places as operators of skill and integrity, but I am, nevertheless, convinced that I could not have been fully understood by them, for I am well satisfied, from the known character for liberality of some of the gentlemen referred to, that if they had used gold foil in this way with the same advantageous results that I have found, they would, long ago, have communicated it to the profession. I could not, of course, suspect them of having availed themselves of its great advantages, and of designedly, during a period when there was the freest interchange of ideas between liberal members of the profession, withholding this valuable mode of operating from their fellow practitioners. I can only come to the conclusion, then, that I have not been clearly understood, and in several instances, a personal interview with some of these gentlemen, has proved this to be the case.

There is, indeed, another view to be taken of the matter, it is this: The modes of practice, and the capabilities of different operators, differ so very widely, that one may find that he can accomplish with gold foil, as it comes from the manufacturer, what another would not attempt, so dim would be his prospect of success. I am free to confess, that I have in years past seen many operations with gold foil, used in the ordinary way, superior to any of the results I have been able to reach with all the labor and time I have been willing to bestow—and I have never been sparing in these essentials of good dental operations. Why it was so, or what was the difference in their methods of manipulation and mine, I have never, in conversation, or in any other way, been able to discover. It is more than probable that this superiority consists in those undefinable qualities which nature gives to some men, enabling them with ease to bring into actual existence the ideals in their own minds; that remarkably prompt and harmonious sympathy between the mind and the hands which is never acquired, if it is not born with the individual. I am quite willing to acknowledge, that with this facility I am endowed in but a limited degree, and that all the good results I have reached, if any, in my profession, has been by hard, patient labor, in the face of this serious natural disability. It may be, then, and it probably has been, lack of skill as an operator which has rendered me dissatisfied with the appliances of our profession, as I found them, and to have led me into the habit of looking anxiously about for the means of getting rid of the difficulties by which I found myself surrounded. For my own use, as I have repeatedly stated, I found the best gold foil defective. I desired something which would better supply my wants, In looking around me I found a great many honest and faithful fellow practitioners in the same category with myself. My earnest desire and effort then was, to find some material which would give better results with less skill, even if it should demand more time and labor. It was with this feeling that I eagerly took hold of sponge gold, as it gave promise of enabling us to get over some of the most perplexing difficulties attending the use of gold. With the same feelings I have turned to the use of gold in the manner I have proposed as a still further advance in the same direction. I have no hesitation in saying that I can obtain better results in the use of either of these materials, in filling teeth, than I can with gold foil as commonly used, possibly at the expense of more time. But I am not at all surprised that gentlemen who can accomplish by the aid of superior skill, with the common material, what I do with greater labor and less skill, should feel no desire to adopt a new method which affords them no advantages. I cannot hope, then, to be of use to these gentlemen, but I feel well satisfied that I may be to that somewhat numerous class of dentists, who, like myself, need all the possible aids to good practice with which they can be furnished. And, whether the system I propose is really new or old, it has certainly never, hitherto, been formally brought to the notice of the profession.

Since the article referred to was published, I have used gold foil in the manner proposed, exclusively, and so have a number of others, with the most beautiful results, and with daily increasing satisfaction.

I have found that it is not necessary to raise the temperature of the foil to a red heat, so that the term, annealed gold, is rather a misnomer. It is no more annealed gold after it is exposed to the slight degree of heat necessary, than it was before; but, for want of a better name, it may probably be well to distinguish it by the name which has appeared to attach itself to it.

It must be premised that all gold foil will not answer for use in the manner proposed. The slight heating to which it is necessary to subject it, does not produce any change in the character of the gold itself. It can only restore it to its original condition. If it is not adhesive after it is annealed by the manufacturers, it will not become so by any degree of heat to which it may afterwards be subjected.

Now, it has been the object of the best manufacturers of gold foil to get rid of the tendency of gold to assume this adhesive character in the course of manufacture. This is accomplished by some means not generally known. It occurs to me, however, that efforts of this kind have, to some extent, been wasted, as by exposure to the air, gold foil, which is very adhesive at the time it leaves the manufacturer, will lose this quality entirely.

How this occurs is a question of interest. It is, of course, well known that gold and other metals become so changed when hammered, as to lose, in a very great measure, their malleability and ductility. But, when exposed to a red heat, such a change is produced in the relations of the particles composing the metal, that its lost properties of malleability and ductility are entirely restored; this application of heat is called annealing.

Experience has shown that although some change in the relations of the particles of which metals are composed, is produced by hammering, or a process analogous thereto, and this change renders the most ductile metal exceedingly brittle, it does not seem that any such change takes place from simple exposure to the atmosphere.

It is found that a slight degree of heat is sufficient to restore the adhesive properties of the surface of gold foil which originally possessed this quality,

If a piece of gold foil originally adhesive, which has been exposed to the atmosphere for a few days, be rolled up, loosely, into a rope and cut into small pieces, it will be found that these pieces may be shaken together without adhering to each other. But if they are laid upon a sheet of thin metal and held for a few minutes over the flame of a lamp, or heated slightly in any other way, the pieces, by their own weight, will adhere to each other so closely as to be separated with difficulty.

Now, it is clear in this case, that the foil -by simple exposure to contact with the air, does not undergo any change analogous to that produced in the metal by hammering. It does not require annealing to restore it to its orginal condition, but the application of a slight degree of heat, not enough, as already intimated, to produce a change in the relations of the particles of which it is composed. But this statement has been questioned; it is supposed, as I have heard it urged, that the thin plate of metal composing the foil, is likely to undergo a change similar to that produced in a larger piece by hammering, simply by handling, and the presure to which it becomes accidentally subject. But if these very same pellets, which have become so adhesive under the influence of slight heat, are separated and allowed to remain until the next day, all their adhesive properties will be gone, and no ordinary pressure will make them unite. If they are again heated, as directed above, they again become as adhesive as before.

A change, then, appears to have taken place upon the surface of the metal. What is this change?

Some years ago, while making a series of experiments in electro-metallurgy, I found that the galvanic deposit could not be made to adhere to a metallic plate, which had been exposed to the air for twenty-fours; but this plate, if warmed, was at once so changed in condition, as readily to receive the metallic desposit, which adhered to it. Indeed this was one of the means proposed to prevent the adhesion of a metallic deposit, when it was desired to take a metallic copy of a certain thing.

In Smee's "Electro-Metallurgy," (pp. 109 and 18, English ed., 1851,) this fact is attempted to be accounted for by supposing that a film of atmospheric air is deposited on the surface of the plate, and that this is dissipated by the application of heat. Whether this theory is correct or not, it seems evident that it is the surface only that is affected.

It is evident, then, that gold to be used advantageously in this way, must, when first made, be adhesive. And this, unquestionably, is the reason of the great differences to be observed in gold of different manufacturers, when attempted to be used in this way. I have tried some foil, unquestionably pure and good, which could not be worked in this way at all, and other specimens which could be used with very trifling advantage.

The foil I first used in this way, is that sold by Jones, White & McCurdy. It worked better than any I had then met with and I still find it, in use, as good as any other. Some specimens of Morgan's gold were then put into my hands, prepared to be used in this way, it worked equally well with that mentioned above. Abbey & Son prepared, at my suggestion, some foil to be used in this way, and it possesses the same fine quality. Their customers, who feel disposed to try the use of foil in the way I am describing, will find it necessary to state that fact when they order, as his gold, sold for ordinary use, does not possess the necessary adhesiveness.

I have not tried, to any extent, the gold of any other manufacturer, although I am assured by all that there is no difficulty in making gold as adhesive as can be desired.

In order to use gold successfully in this way, everything depends upon the instruments employed and the manner of manipulation. The instruments are simple and easily made, and the manipulations are not difficult. All that is necessary is strict attention to certain essential points.

In the first place the gold must possess the quality I have described, and I would advise any operator who is disposed to make trial of gold in this way, to procure gold of one of the manufacturers named above, stating the purpose for which it is wanted.

There are two methods in which gold may be prepared for use in the manner proposed. It may either be used in the form of pieces cut from a rope, or the sheet may be cut up or torn into small pieces, without folding or rolling it up.

1. In preparing the pellets, the sheet of No. 6 foil should be cut into strips according to the size of the cavity. No definite directions can be given upon this point, as experience alone can teach an operator how much he may want for a particular cavity. I generally cut my sheet into three strips. These strips should be rolled up loosely, *as loosely as possible*, laid upon a thin piece of platinum or other thin metallic plate, and heated over the spirit lamp. The degree of heat is unimportant, if continued long enough, but it will be sufficient if the platinum is allowed, directly under the rope of gold, to reach a dull red heat. The roll is then cut up into pieces of a suitable size. The direction to roll the strip up loosely is important, as many persons who have attempted to use gold in this way, have failed because they have rolled it too tightly.

The instruments used for this gold should terminate in two or more sharp points. The most convenient for general use are those made with two, three and four points.

The curve given to the shank of the instruments must, of course, be determined by the operator, to suit the special case.

The points must be sharp ; they cannot be made so without a suitable file, and the best file for the purpose is that known to watchmakers as a pivot file. It has a sharp, knife edge, and is admirably adapted to the purpose.

The temper given to those instruments must be somewhat harder than that of ordinary plugging instruments, a little harder than a spring temper. It is difficult to state in writing the exact temper to be given; this can best be ascertained by a few trials. The instruments should be as hard as they can be made without rendering them so brittle as to break with necessary use.

In using gold in this form, the greatest difficulty to be encountered is to fix the pieces first put into the cavity; after a part of the filling becomes firmly fixed in any part of the cavity the rest of the operation is very easy. I have, for a long time, been accustomed to hold a small instrument in my left hand, and with it keep in place my first pieces of gold until they become fixed. Sometimes, however, this is extremely difficult, and, at times, impossible. Dr. Louis Jack suggested a very useful way ot getting over the difficulty referred to. He proposed to drill two small holes about a twelfth of an inch deep in a part of the cavity where there is no danger of reaching the pulp, begin his operation at this point, as there is no difficulty in getting the gold to remain in such small cavities, and build the filling up from these two points.

After the first pieces are fixed, pellet after pellet is taken up with the point of the instrument used, carried to the desired place, fixed by pressure against the gold previously put into the cavity, and condensed, first with large and then with small instruments, taking care to carry it against the sides of cavity. Thus going on, adding piece after piece, until the cavity is filled.

It is essential in using gold in the manner proposed, that the cavity should be kept dry; if the slightest quantity of moisture finds its way upon the surface of the filling, the operation for the time is at an end. I have seen cases where an apparently good filling has been made under the saliva; but I cannot regard such a filling as so secure and reliable as when no moisture interposes itself between the layers. If in performing a long operation it is found that the saliva is encroaching upon your filling, burnish the surface, which, if you have manipulated properly, ought to be condensed. Allow your patient to void his mouth of saliva, and then recommence your operation. Dry the filling and the parts near it, then scrape the surface so as to remove any moisture which may have become confined about the surface. More gold may then be added, as well as if no moisture had reached the filling; and so more and more may be added, until a satisfactory filling is made.

2. I prefer, however, to use gold, not in the form of pellets, but the single sheet, neither folded nor rolled, but cut or torn into pieces of suitable size, after the sheet has been heated on a piece of platinum or any other kind of thin metal plate.

The instruments in this case differ from those just described. They should have a somewhat broader surface, cut with as many points as can be got upon it. The serrations should not be so deep as in the instruments for pellets. I have some instruments with eight, ten, twenty and forty points.

After the gold is heated I generally hold it in a pair of spring forceps, as it should not be touched with the fingers, and tear it in pieces of a suitable size with a sharp instrument. As I am not now writing for novices in the profession it is unnecessary to go into detail; every operator will soon learn, by experience, what size his pieces will need to be for any cavity in hand.

After drying the mouth, the cavity, and the parts about the tooth to be filled, with great care, a piece of the gold is carried into the cavity. If I can hold it in place until I can fix a sufficient quantity to make a beginning for my filling, I prefer to do so. If I cannot conveniently do this, I adopt the method, where I can, of Dr. Jack, mentioned above. After the gold becomes fixed in any part of the cavity, the rest of the operation is easy. The thin pieces of gold are taken up and condensed thoroughly in thin layers with the broad, sharply serrated instruments, the operator being careful not to gather any considerable quantity under his instrument while he is condensing his gold. Care must be taken, too, to carry the gold against the sides of the cavity as the filling progresses. The rapidity with which a large and accessible cavity may be filled, when the gold is used in the manner described is surprising. But when the cavity is filled, using ordinary pressure with the instruments described, the gold will be found so dense as to be unyielding under small and sharp instruments. The reason of this is easily seen; the condensation of the gold in thin layers has been effected, and it is easy to understand how a few thin layers of gold over a broad surface, may be brought as compactly together with comparatively slight force, as a large number of layers such as are to be found in a pellet, with much greater force and with much smaller instruments.

I have been in the habit of using No. 6 in my operations. In thin, frail cavities, No. 4. No. 8 has been proposed by Prof. Townsend, and no doubt will facilitate the operation in large cavities by using it in a single strip.

I am well aware of the difficulty of describing, in a manner to be clearly understood, any method of manipulating. A few demonstrations will do more to elucidate a practical subject than pages of explanation. I should suppose, however, that the suggestions I have made, vague as they may be, will enable any experienced operator to ascertain for himself the amount of value of the method of operating which I have been describing.

In this vicinity a number of operators have adopted this method with so much satisfaction and success, that they declare they could not fill the cavity of a tooth, at all satisfactorily to themselves, in the manner they had been operating for years before. I have seen first class operations from the hands of men who had had no experience in our profession—such operations as certainly could not have been performed, in the usual way, except after years of practice.

I feel impelled to urge, strongly, every one in the profession to give this method a fair and thorough trial, and I am satisfied that when once understood, it will be generally adopted, to the almost entire exclusion of other methods of using gold foil. I have never yet known an operator, after he has once become fully aware of its advantages, who has abandoned its use.—*News Letter.*

